# Linarine inhibits inflammatory responses in dry eye disease mice by modulating purinergic receptors

**DOI:** 10.3389/fimmu.2024.1463767

**Published:** 2024-09-30

**Authors:** Pei Liu, Pengfei Jiang, Kang Tan, Yunfeng Yu, Genyan Qin, Tingting Liu, Sainan Tian, Jun Peng, Qinghua Peng

**Affiliations:** Hunan Provincial Key Laboratory of Traditional Chinese Medicine (TCM) Diagnostics, Hunan University of Chinese Medicine, Changsha, Hunan, China

**Keywords:** dry eye disease (DED), linarine, purinergic receptors, inflammatory responses, cornea

## Abstract

**Background:**

Linarine is a natural chemical component widely found in Buddleja officinalis Maxim., Chrysanthemum indicum L., Mentha canadensis L., and other medicinal plants. Modern pharmacological studies have shown that linarine with good anti-inflammatory and antioxidant activities can inhibit the proliferation and induce apoptosis of many kinds of tumor cells. Moreover, linarine showed protective effect on the liver, kidneys, and other organs.

**Methods:**

Inflammation model of human corneal epithelial cell (HCEC) was constructed using NaCl induction, and cytotoxicity was detected by the CCK8 assay. The levels of inflammatory factors tumor necrosis factor-α (TNF-α) and interleukin 1β (IL-1β) were measured using Enzyme-linked immunoassay (ELISA). Chronic painful stimulation (tail clamping) in combination with Benzalkonium Chloride Solution drops in a desiccator established a mouse model of dry eye disease (DED). The following parameters were recorded: body mass, anal temperature, tear secretion, tear film rupture time, and corneal fluorescein staining. The levels of inflammatory factors mitogen activated protein kinase (MAPK), nuclear factor kappa-B (NF-kB), c-Jun N-terminal kinase (JNK), IL-1β, Interleukin 18(IL-18), A2A, A3, P2X4, P2X7, P2Y1 were measured by using immunofluorescence (IF) staining.

**Results:**

Linarine can inhibit the secreation of TNF-α, and IL-1β in HCECs. Linarine prolonged tear film rupture time, promoted tear secretion, repaired corneal damage, and reduced the levels of inflammatory factors of MAPK, NF-kB, JNK, IL-1β, IL-18, and modulated the levels of the purinergic receptor.

**Conclusions:**

Linarine is effective in treating dry eye in mice by inhibiting purinergic receptors-mediated inflammatory response.

## 1 Introduction

Dry eye is a common ocular surface disease characterized by insufficient tears or excessive tear evaporation, resulting in the inability to maintain normal lubrication and wetness of the ocular surface, which can cause ocular discomfort, blurred vision, and corneal damage (1). The etiology of dry eye is complex and varied, usually including age-related factors, sex hormone changes, autoimmune diseases, environmental factors, and long-term use of electronic devices. In recent years, the incidence of dry eye has been on the rise, seriously affecting the life quality of patients (1, 2). Therefore, the search for effective treatments and interventions has become an important direction in ophthalmologic research.

Inflammatory response plays a key role in the development and progression of dry eye (3). Significant increase in the infiltration of inflammatory cells on the ocular surface and the release of tumor necrosis factor-α (TNF-α), interleukin-1β (IL-1β), and so on was found in the patients with dry eye. These inflammatory factors not only destabilize the tear film but also aggravate the inflammatory response of the ocular surface, forming a vicious circle (4). Therefore, inhibiting the inflammatory response on the ocular surface is considered to be an effective strategy for the treatment of dry eye.

Purinergic receptors, which widely distributed on the ocular surface, such as the cornea, conjunctiva, and lacrimal gland, play important roles in a variety of physiological and pathological processes, including nerve conduction, immune regulation, and inflammatory response. This kind of receptor mainly include P1 and P2 receptors and have various subtypes, among which purinergic A2A, A3, P2X4, P2X7, and P2Y1 receptors play important regulatory roles in the inflammatory response of dry eye (5). For example, the P2X7 receptor, an ATP-gated cation channel, plays an important role in the inflammatory response to dry eye. Activation of the P2X7 receptor promotes the release of inflammatory factors and exacerbates the inflammatory response on the ocular surface (5–7). Therefore, modulation of purinergic receptor activity may be a novel strategy for the treatment of dry eye.

Linarine, a natural compound extracted from the plant Rubiaceae, possesses a variety of biological activities, including anti-inflammatory, antioxidant, hepatoprotective, and neuroprotective effects. Studies have shown that linarine can inhibit inflammatory responses through various pathways, such as inhibiting the nuclear factor kappa-B (NF-κB) signaling pathway and reducing the release of inflammatory factors (8). In addition, linarine can exert its anti-inflammatory effects by regulating other signaling pathways, such as nuclear factor kappa-B (MAPK), Toll-like receptor 4 (TLR4), etc (9).

**Scheme 1:**
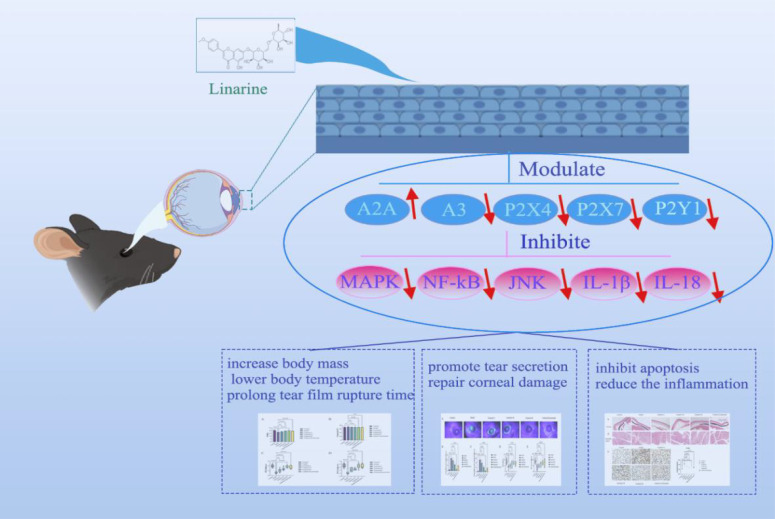
Linarine’s applicability in therapy for DED mice.

This study aims to investigate the role of linarine in dry eye and its mechanism, especially its modulation of purinergic receptors (**Scheme 1**). Specifically, we investigated whether linarine can improve dry eye symptoms by modulating purinergic receptors and inhibiting inflammatory responses on the ocular surface. First, we investigated the effects of linarine on Human corneal endothelial cells (HCECs) through *in vitro*. Second, we utilized an animal model of dry eye to evaluate the effects of linarine on dry eye symptoms, tear secretion, purinergic receptor expression, and ocular surface inflammatory response, to reveal the specific mechanism of linarine’s modulation on purinergic receptors.

## 2 Material and methods

### 2.1 Cell lines

The cell lines present in this study were obtained from Guangzhou Saiku Biotechnology Co., Ltd. Human corneal endothelial cells (HCECs) were cultured at 37°C with 5% CO2 and treated with 94 mM NaCl for 24 h were used to simulate the dry eye model. DMEM/F12 basic medium, 5% fetal bovine serum 10ng/mlhEGF, 25mM insulin, and 1% double antibiotic solution were used to prepare cell culture medium. The cell fusion degree was 80%-90% for passaging. Cell transmigration: discard the cell culture medium, add PBS, gently shake to clean, add 2ml 0.25% Trypsin-EDTA, place in the cell culture incubator to digest for 2min, observe the cells crumple and become round, add cell culture medium to terminate the digestion, use a pasteurized tube to blow the cells and detach them from the wall of the bottle, transfer the cell suspension to 15ml centrifugal tube, 1000r/min, centrifuge for 5min, discard the supernatant, add cell culture medium to blow and mix until no clumps, according to 1:3 transmigration, add 5ml of cell culture medium respectively and freeze. 5min, discard the supernatant, add cell culture medium and blow and mix until no lumps, according to the 1:3 passaging, respectively, add 5ml of culture medium to continue cultivation.

### 2.2 Animals

Sixty SPF-grade female C57BL/6 mice were randomly divided into 6 groups (n=10), control group, model group, linarine-L group (12.5 mg/kg-d), linarine-M group (25 mg/kg-d), linarine-H group (50 mg/kg-d), and Sodium Hyaluronate group. The mice in the control and model groups were gavaged with deionized water at 1 mL/100g. Mice in the Sodium hyaluronate group were given eye drops of Sodium hyaluronate. This treatment lasted for 2 weeks. Establishment of dry eye in mice by 28 d of benzalkonium chloride eye drops combined with chronic pain stimulation (tail pinching) in a desiccator. The mouse ocular surface was treated with 0.2% benzalkonium chloride solution for eye drops, 5 times a time in μL, 2 times a day, for a total of 4 weeks (10). All operations conformed to the ethical requirements of animal experiments at Hunan University of Chinese Medicine (ethics number: LL2022092104). Experimental protocols involving animals and their care were approved by the Institutional Animal Care and Use Committee of Hunan University of Chinese Medicine, and all animal experiments were carried out in strict accordance with the Guide for the Care and Use of Laboratory Animals of the National Institute of Health.

### 2.3 Reagents and chemicals

Linarine (Beijing Soleberg Technology Co., Ltd., IB0570), Benzalkonium chloride (Sigma, USA, 12060), Sodium hyaluronate eye drops (Santen Pharmaceutical Co.,Ltd., H20171192), isoflurane (Hebei Yipin Pharmaceutical Co., Ltd., H19980141), phenol red cotton thread (Tianjin Jingming New Technology Development Co., Ltd., 20192160086). Tumor Necrosis Factor α (TNF-α) ELISA Kit (SEKH-0047), Interleukin 1β (IL-1β) ELISA Kit (SEKH-0002), A2A antibody (K009523P), A3 antibody (K008096P), P2X4 antibody (K004709P), P2X7 antibody (K002032P), P2Y1 antibody (K005693P), MAPK antibody (K001612P), NF-kB antibody (K003592P), c-Jun N-terminal kinase (JNK) antibody (K009566P), IL-1β antibody (K009661P), interleukin-18 (IL-18) antibody (K004534P) were acquired from Beijing Soleberg Technology Co., Ltd. (Beijing, China).

### 2.4 Detection indicators and methods

#### 2.4.1 Cell viability assay

The cell viability was detected using CCK8 cell counting kit, and the cells in each group were treated with different media for cells for 24 h. The cells were incubated with CCK8 reagent for 2 h. The cells were incubated for 2 h with PBS and CCK8 reagent. Subsequently, the absorbance was measured at 450 nm using an enzyme marker.

#### 2.4.2 Enzyme-linked immunoassay

Sample Acquisition: HCECs supernatant: 24-well plate laying 24h after the administration of the drug, 2 hours after the administration of the drug, in addition to the control, Model and the administration of 94 mM NaCl for induction, 24h after the supernatant centrifugation (1000 rpm, 10min), the supernatant was collected -20/-80°C storage to be measured.

Dilution: HCECs supernatant IL-1β factor was not diluted; TNF-α was diluted 50 times, and control group was not diluted.

ELISA kit operation: The specific operation procedure is strictly referred to the ELISA kit manual, IL-1β factor using the standard curve 18.746x2 + 168.48x - 0.0163, TNF-α factor using the standard curve y = 18.779x2 + 224.32x - 12.449. 3 replicates were set up for each group, and the OD value of each well was measured at 450 nm. The absorbance of the samples was substituted into the standard curve equation to obtain the concentration of the samples. The results were compared between groups using the statistical test ANOVA to determine if the differences between groups were statistically significant.

#### 2.4.3 Weight

After two weeks of drug treatment, the mice were weighed using a T1000 electronic balance.

#### 2.4.4 Temperature

After two weeks of drug treatment, insert the probe of the mouse anal temperature detector into the anus of the mouse and measure the anal temperature, enter the anus completely and hold it for at least 10 seconds and record the temperature.

#### 2.4.5 Schirmer I test

Phenol red cotton thread was used to determine tear secretion in mice. A cotton swab was gently immersed into the conjunctival sac to remove excess tears. Under no anesthesia, the lower eyelids of mice were gently pulled downward, the phenol red cotton thread was picked up with forceps, and a 1-mm portion of the thread was placed on the conjunctiva for 30 seconds at a position about one-third of the way between the lower eyelid and the canthus, and the length of the phenol red cotton thread was measured by a ruler in millimeters (mm) under a microscope to wet the red colouring length of the thread. Each eye was tested 3 times (10).

#### 2.4.6 Fluorescein breakup time

1 μL of 1% fluorescein sodium solution was placed into the conjunctival sac of the mouse and excess liquid was aspirated with blotting paper. 10 seconds after observation under a cobalt blue light slit-lamp microscope, the time from the beginning of the transient to the first dark spot on the cornea was considered However, this procedure was repeated 3 times per eye and averaged (10).

#### 2.4.7 Corneal fluorescence staining

1 μL of 1% sodium fluorescein liquid was introduced into the conjunctival sac of rats, and blotting paper was used to aspirate the excess liquid for cobalt blue light slit lamp microscopy. The cornea was divided into 4 quadrants, with each quadrant scored from 0 to 4, and the total score was 16. Score 0: no staining; score 1: punctate staining, but less than 30; score 2: more than 30 spots of staining, but not diffuse; score 3: obvious diffuse staining but no patchy staining; score 4: patchy staining. The scores of the 4 quadrants were added together to obtain the corneal score.

#### 2.4.8 Terminal deoxynucleotidyl transferase dUTP nick end labeling to detect apoptosis

Lacrimal glands were fixed with 4% paraformaldehyde, embedded in paraffin, sectioned and prepared for tunnel staining. (a). Sections are dewaxed in xylene for 5-10 min. Replace with fresh xylene and dewax for another 5-10 min. Anhydrous ethanol for 5 min. 90% ethanol for 2 min. 70% ethanol for 2 min. distilled water for 2 min. (b). Dropwise addition of 20 μg/ml DNase-free Proteinase K. 20-37°C for 15-30 min. Wash 3 times with PBS. (c). Incubate in 3% hydrogen peroxide solution prepared in PBS (3% H2O2 in PBS) for 20 min at room temperature to inactivate endogenous peroxidase in the sections. Subsequently, the sections were washed three times with PBS. The samples were incubated for 60 min at 37°C away from light. (d). 50 μL of biotin labelling solution was added to the samples and incubated for 60 min at 37°C away from light. (e). The samples were washed once with PBS, 0.1-0.3 ml of labelling reaction termination solution was added dropwise, and the samples were incubated for 10 min at room temperature. (f). The samples were washed 3 times with PBS. Add 50μL of Streptavidin-HRP working solution to the sample and incubate for 30 min at room temperature. Wash 3 times with PBS. (g). Add 0.2-0.5 ml of DAB color development solution dropwise and incubate for 5-30 min at room temperature. h. Observe under a microscope and capture images.

#### 2.4.9 Hematoxylin and eosin staining

HE staining was performed on the lacrimal gland, cornea, liver, heart, spleen, lung and kidney to observe the cytological morphology. The samples were placed in 4% paraformaldehyde fixation solution for 15~20 min, and then the excess tissue was cut off, dehydrated with gradient alcohol, transparent with xylene, and buried in wax. (a). Paraffin section dewaxing to water: the sections were sequentially treated with xylene I for 20 min - xylene II for 20 min - anhydrous ethanol I for 5 min - anhydrous ethanol II for 5 min - 75% alcohol drying for 5 min, and washed with tap water. (b). Hematoxylin staining: Hematoxylin staining was done for 3-5 min, hydrochloric acid aqueous solution was differentiated, and the ammonia aqueous solution returned to the blue, and washed with water. (c). Eosin staining: the sections were sequentially dehydrated in 85% and 95% gradient alcohol, and then stained in eosin staining solution for 5min. (d). Dehydration and sealing: the sections were sequentially put into anhydrous ethanol I for 5min - anhydrous ethanol II for 5min - anhydrous ethanol III for 5min - xylene I for 5min - xylene II for 5min for transparency, and then sealed with neutral gum. (e). Motic 6.0 digital medical image analysis system (Shenzhen Yuanheng Technology Co., Ltd., Shenzhen, China) was used to observe the pathological changes of the groups and take pictures.

#### 2.4.10 immunofluorescence staining

(a).The eyeballs were cut into 10-μm slices by a cryosectioner, bake the slices at 60°C for 12 h. (b). Deparaffinization of the slices to water: the slices were first placed in xylene for 20 min, 3 times. Then they were sequentially placed in 100%, 95%, 85% and 75% ethanol for 5 min at each level. then they were rinsed by immersion in distilled water for 5 min. (c). Thermal repair of antigens: the sections were immersed in EDTA buffer (pH 9.0),heated to boiling in an electric oven or microwave oven and then disconnected from the power, and boiled continuously for 24 min, then cooled for 24 min and taken out and cooled to room temperature. After cooling, the sections were washed with 0.01M PBS (pH7.2~7.6) for 3 min x 3 times. (d). Sections were placed in sodium borohydride solution for 30 min at room temperature and rinsed with tap water for 5 min. (e). Sections were placed in 75% ethanol solution for 15s~1 min. (f). sections were placed in Sudan black dye solution for 15 min at room temperature and rinsed with tap water for 5 min. (g). 10% normal serum/5% BSA closed for 60 min. (h). Incubate primary antibody: dropwise add appropriate dilution (1:10-200) of primary antibody, overnight at 4°C. Rinse with PBS for 5 min x 3 times. (i). Incubation of secondary antibody: dropwise addition of 50~100ul anti-rabbit or mouse-IgG labelled fluorescent antibody, incubate at 37°C for 60~90 min, PBS rinse 5 min x 3 times. (j). DAPI working solution stain nuclei at 37°C for 10~20 min, rinse with PBS for 5 min x 3 times. (k). Buffered glycerol to seal the film. (l). Store away from light, or observe under fluorescence microscope. (m). The fluorescence was quantitatively analyzed using Image J.

#### 2.4.11 Statistical analysis

SPSS 26.0 software was used for statistics, and measurement data were expressed as mean ± standard deviation (SD). If they obeyed normal distribution, comparisons between groups were performed using ANOVA, and if differences between groups were statistically significant, two-way comparisons between groups were performed using the LSD test. If they did not obey normal distribution, the Kruskal-Wallis rank sum test was used for comparisons between groups. For multiple group comparisons, ANOVA was used if they obeyed normality and chi-square, and the rank sum test was used for those that did not. *P* < 0.05 was considered statistically significant.

## 3 Results

### 3.1 Linarine reduces the expression of inflammatory factors in HCECs

**Figure 1:**
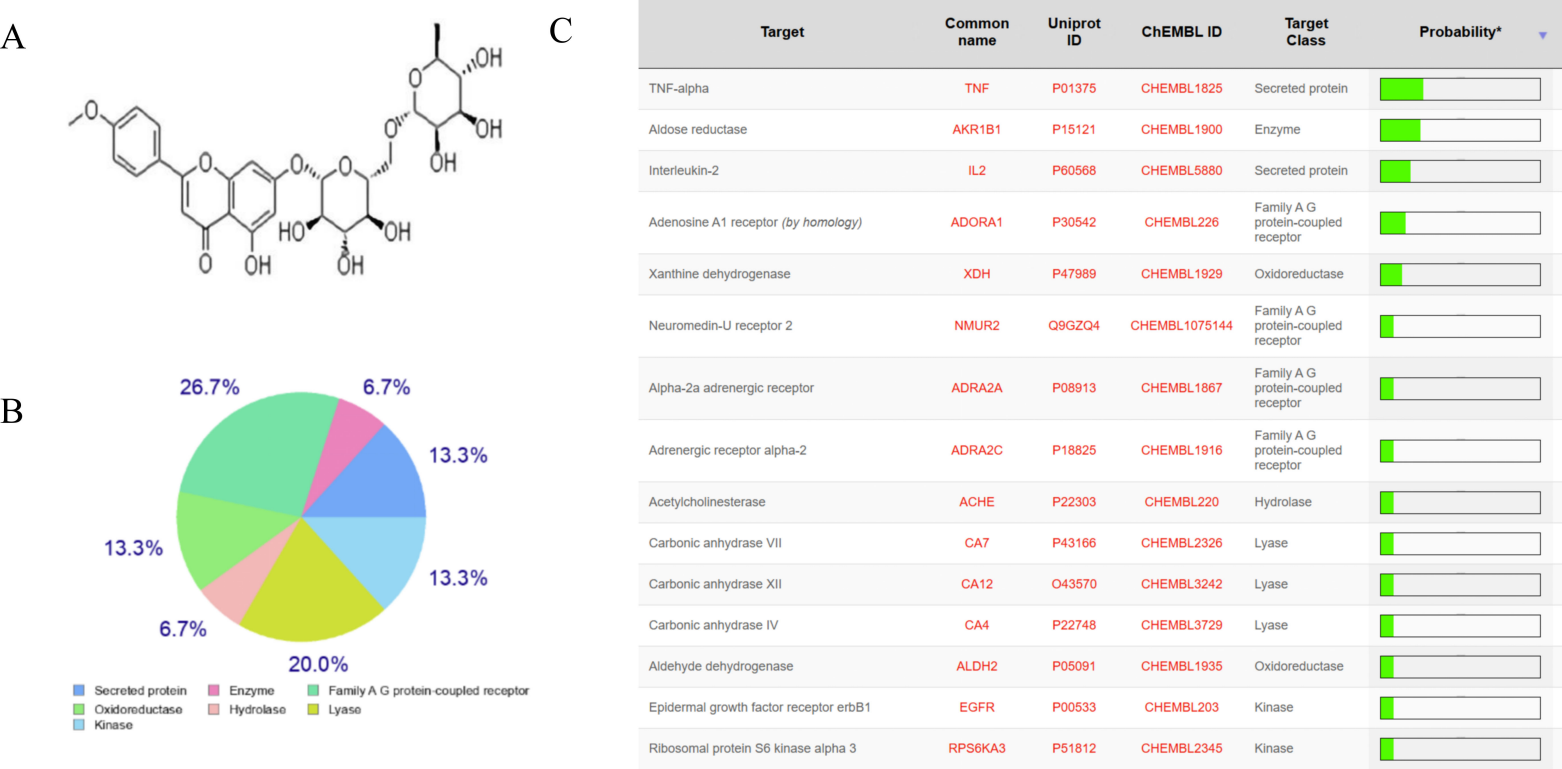
The anti-inflammatory target of linarine. **(A)** Structure of linarine, **(B, C)** The top 15 linarine-related proteins analyzed by Swiss Target Prediction server.

**Figure 2:**
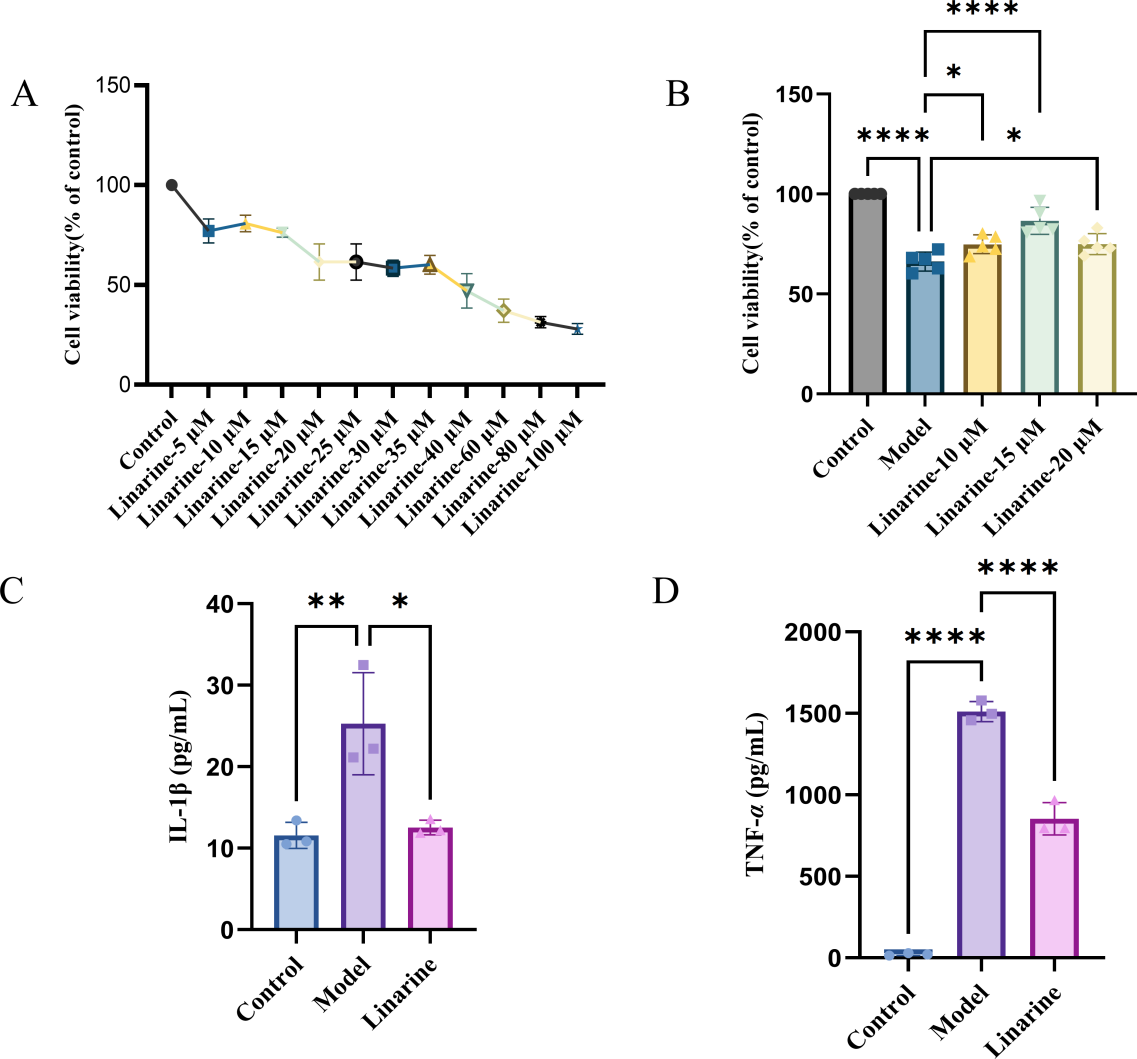
Linarine reduces the expression of inflammatory factors in HCECs. **(A)** Cytotoxicity of the HCECs incubated with various concentrations of linarine for 24 h (n=4). **(B)** The viability of linarine at different concentrations with inflammation model (n=5). **(C, D)** ELISA of TNF-α and IL-1β levels in HCECs. Values are mean ± SD from 3 samples. ns *P* > 0.05, *P < 0.05, **P < 0.01, ****P < 0.0001 compared with model group.

To clarify the effect of linarine on DED, we screened and obtained the top 15 linarine-related active targets from the Swiss Target Prediction database such as TNF, IL, etc. (**Figure 1**). These proteins are extensively involved in inflammatory, immune responses. Then, we examined the effect of linarine in HCECs. As shown in **Figure 2A**, cell viability decreased continuously with increasing monensin concentration. Highest cell survival rate of 86.61% in a Nacl-induced inflammatory model at the presence of 15 μM linarine (**Figure 2B**). Compared with the control group, the level of IL-1β in the model group was elevated on average by 13.7(*P* < 0.01). The level of IL-1β was reduced on average by 12.74 after linarine treatment (*P* < 0.05). Compared with the control group, the level of TNF-α in the model group was elevated on average by 1489.06(*P* < 0.0001). The level of TNF-α was reduced on average by 657.9 after linarine treatment (*P* < 0.0001) (**Figures 2C, D**).

### 3.2 Linarine regulates body weight and body temperature in mice and improves tear film stability

**Figure 3:**
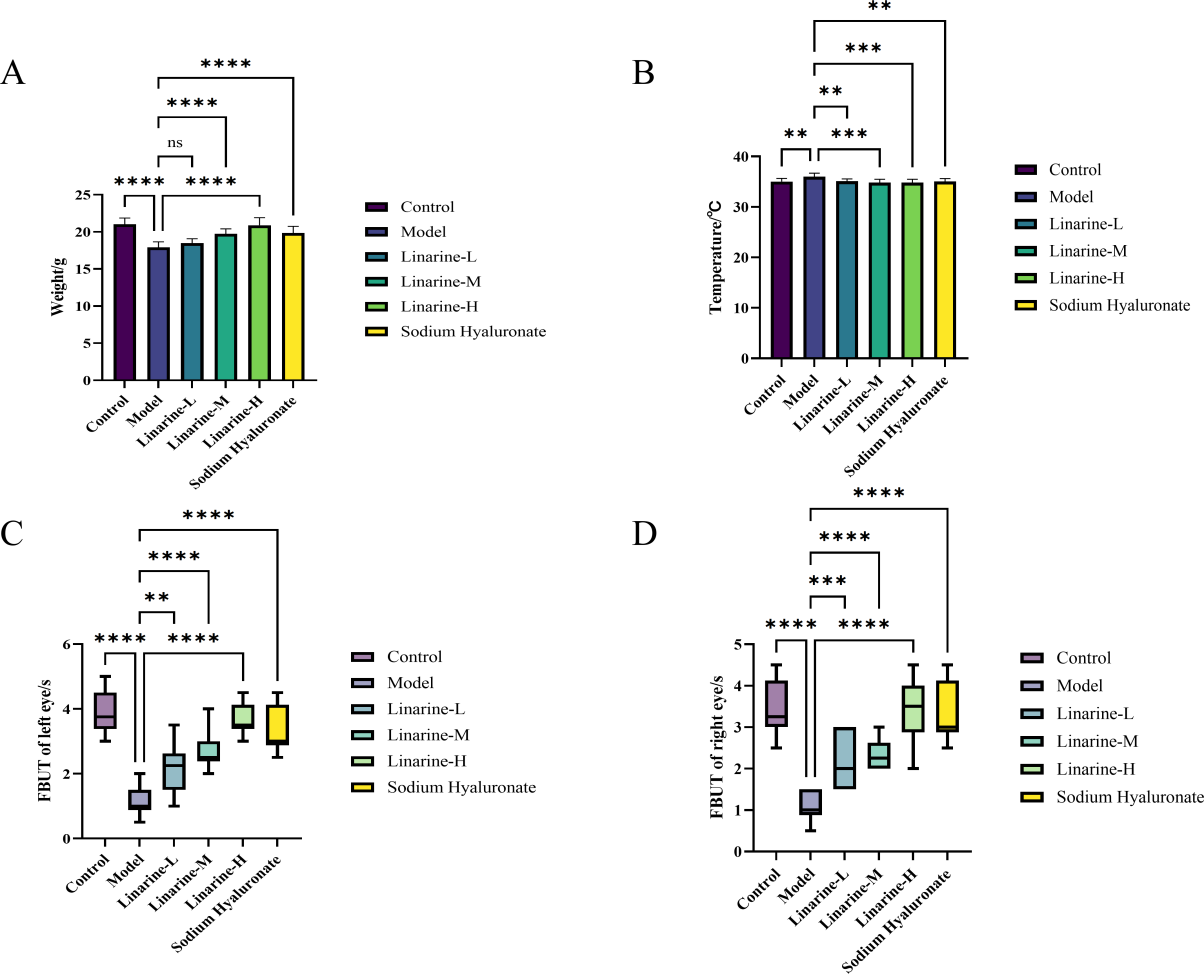
Effect of linarine on temperature **(A)**, weight **(B)**, and FBUT **(C, D)** of different experimental groups. Values are mean ± SD from 10 samples. ns *P* > 0.05, **P < 0.01,***P < 0.001, ****P < 0.0001 compared with model group.

We observed a significant weight gain effect in the linarine-treated mouse model. The mice in the experimental group gained an average of 2.96 g of body weight after treatment with a high dose of linarine (*P* < 0.0001) (**Figure 3A**). In addition, linarine significantly reduced the body temperature of the mice, with an average decrease of 1.18 degrees Celsius in the experimental group after high-dose linolenine treatment (*P* < 0.001) (**Figure 3B**). These results suggest that linolenine may regulate body weight and body temperature in mice by affecting metabolic or nervous system activities.

In the linarine treatment, we observed a significant increase in tear stability in the treated group of animals. Analyses showed that compared to the model group, the left tear film rupture time in the linarine-H group improved by an average of 2.6s (*P* < 0.0001), and the right tear film rupture time improved by an average of 2.35s (*P* < 0.0001) (**Figures 3C, D**).

### 3.3 Linarine increases tear secretion and improves tear film stability

**Figure 4:**
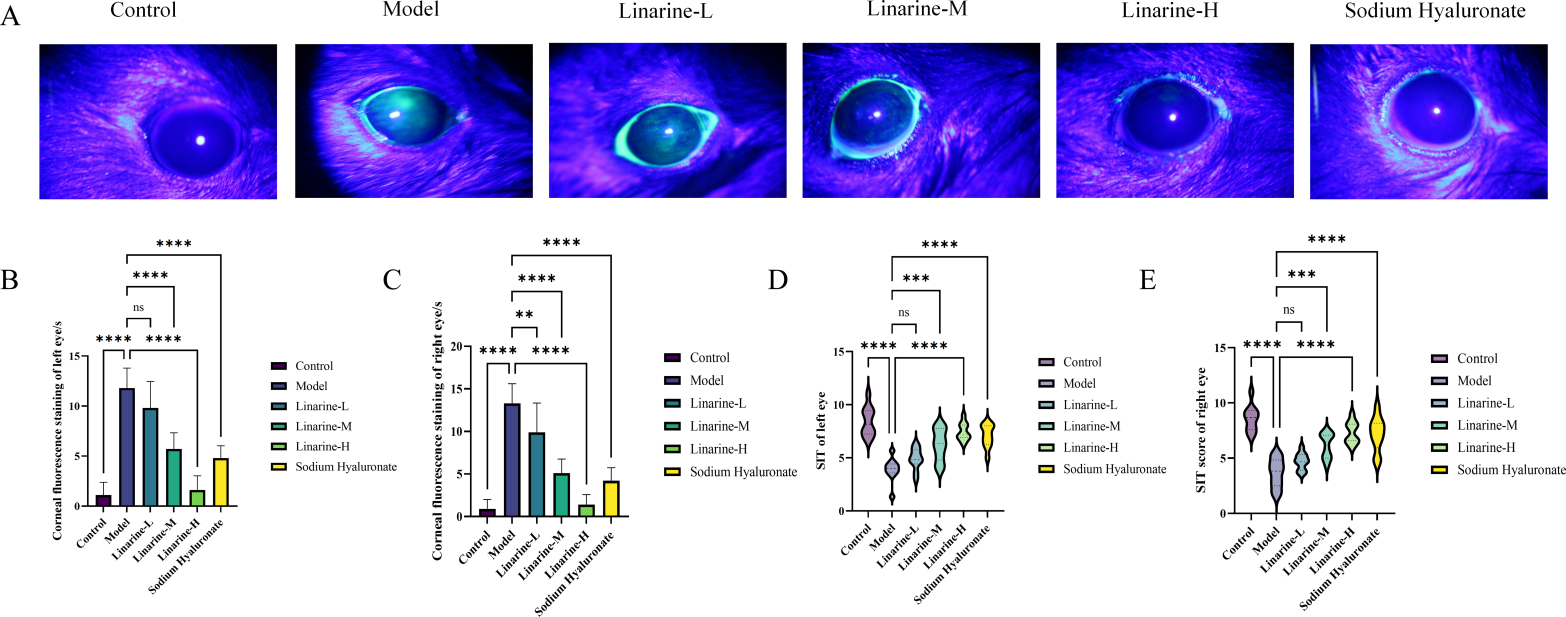
Effect of linarine on fluorescence staining **(A)**, Corneal fluorescence staining score **(B, C)**, and SIT **(D, E)** of different experimental groups. Values are mean ± SD from 10 samples. ns *P* > 0.05, **P < 0.01,***P < 0.001, ****P < 0.0001 compared with model group.

We investigated the effect of linarine on corneal fluorescence staining in a mouse model. The results showed that the intensity of corneal fluorescence staining was significantly attenuated in the linarine-treated group, displaying a significant reduction compared with the model group (*P* < 0.001) (**Figures 4A-C**). Linarine may modulate corneal fluorescence staining by improving the lubricity of the corneal surface or by protecting the cells of the epithelial layer. We investigated the effect of linarine on tear secretion in an animal model. Tear secretion was measured using Schirmer’s test after daily administration of linarine treatment, which showed a significant increase in tear secretion in the treated group of animals. Tear secretion in the left eye increased by 3.57 mm and in the right eye by 3.67 mm in the linarine-H group compared to the model group (*P* < 0.001) (**Figures 4D, E**).

### 3.4 Linarine promotes the restoration of corneal and lacrimal gland morphology

**Figure 5:**
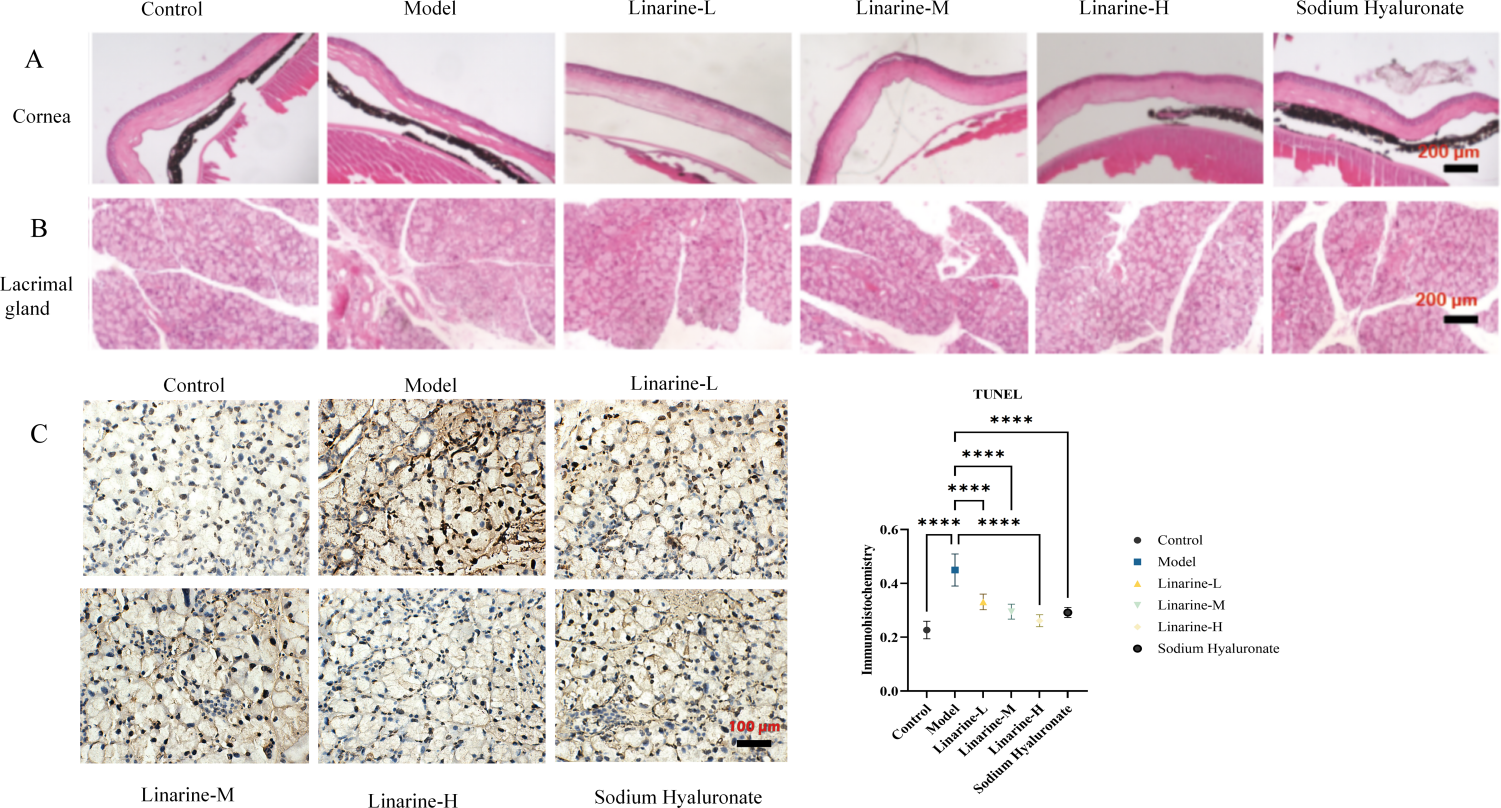
Effect of linarine on cornea cell apoptosis **(A)**, lacrimal gland **(B)**, and TUNEL staining result **(C)** in each group. Values are mean ± SD from 3 samples. ****P < 0.0001 compared with the model group.

The effect of linarine on the structure of mouse cornea and lacrimal gland tissue was analyzed by HE staining. Morphological changes in the corneal lacrima and gland tissue were observed after treatment with linarine. In the normal group, the corneal surface was smooth, with 4-6 layers of epithelial cells in the corneal tissue, clear layers, tightly arranged, and neat corneal stroma; in the model group, the cornea was accompanied by shedding of cells of the upper layer of the epidermis, disordered arrangement of stromal cells, and edema; the corneal sections of the linarine groups of mice showed a reduction in the shedding of the epithelial cells and edema of the stromal layer compared to the corneal sections of the model group of mice (**Figure 5A**). The size of the lobules of the lacrimal gland in the normal group of mice was consistent, and the follicular cells were conical, with many cytoplasmic zymogen particles and no inflammatory cell infiltration; the follicular cells of the model group were increased in size and disordered in arrangement, with a large number of inflammatory cell infiltration and increased manifestations of neovascularization; the vacuoles of the follicular cells of the linarine groups were reduced, and the infiltration of the inflammatory cells was reduced, and neovascularization was reduced (**Figure 5B**). The linarine-treated group showed a tighter cellular arrangement and clearer nuclear staining compared to the control group. These results imply that linarine may improve the pathology of ocular diseases by promoting the health and function of cornea and lacrimal gland tissues. The linarine groups showed reduction in apoptosis of lacrimal gland cells when observed under HE staining. Lacrimal gland tissues in the linarine treated group showed more cells remaining intact and reduced visible apoptosis as compared to the model group (*P* < 0.0001)(**Figure 5C**).

### 3.5 Linarine regulates purinergic receptor expression

**Figure 6:**
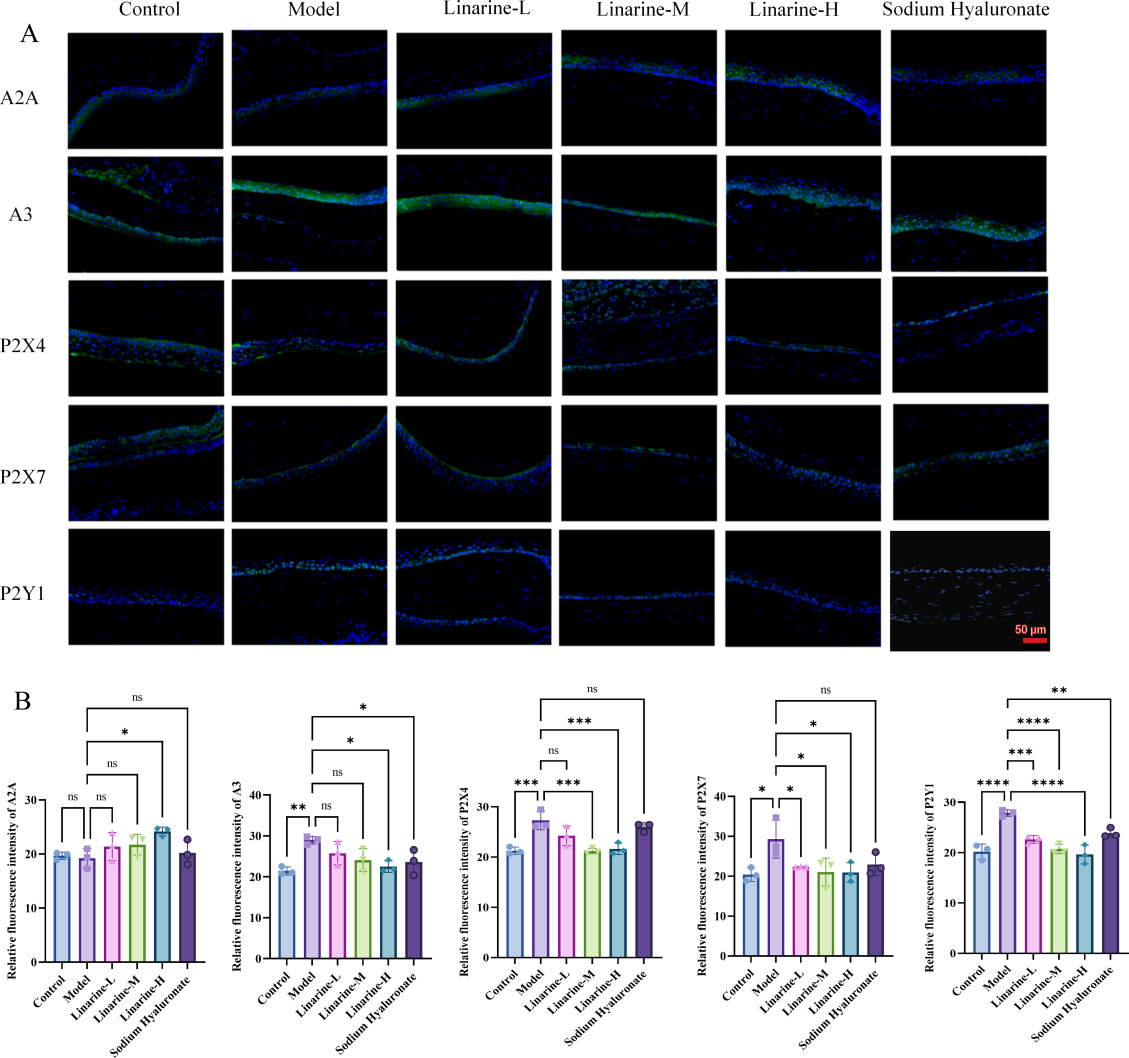
Effect of linarine on IF of purinergic receptor-related proteins. **(A)** IF of A2A, A3, P2X4, P2X7, and P2Y1 and **(B)** Fluorescence quantitative analysis. Values are mean ± SD from 3 samples. ns *P* > 0.05, *P < 0.05, **P < 0.01,***P < 0.001, ****P < 0.0001 compared with model group.

Compared with the control group, there was no statistically significant difference in the level of A2A in corneal epithelial cells in the model group, while the expression of A3, P2X4, P2X7, and P2Y1 was elevated by an average of 7.508, 6.040, 8.897, and 7.689. After linarine treatment, the average expression level of A2A in the linarine-H group increased by 4.968, while the average expressions of A3, P2X4, P2X7, and P2Y1 decreased by 6.444, 5.646, 8.352, and 8.252, respectively (**Figures 6A, B**). The A2 receptor is normally anti-inflammatory and reduces inflammation by modulating the immune response. Its elevation may indicate that linarine treatment is attempting to enhance the anti-inflammatory mechanisms and alleviate the inflammation associated with dry eye. A3 receptors are typically associated with pro-inflammatory effects, and their lowering could help reduce inflammation in dry eye and improve corneal health. P2X4 receptors are associated with pain and inflammatory responses, and lowering their expression could help reduce ocular discomfort and inflammation. Reduced expression of P2X7 receptors helps to reduce inflammation and protect corneal cells, improving symptoms of dry eye and corneal health. P2Y1 receptors are involved in the regulation of inflammation and cellular function, and reduced expression may reduce inflammation and regulate cellular function. By IF analysis, we observed significant changes in the expression levels of specific purinergic receptors after linamarine treatment. This suggests that linarine may affect relevant cell signaling pathways by inhibiting the expression of purinergic receptors.

### 3.6 Linarine reduces the inflammatory response

**Figure 7:**
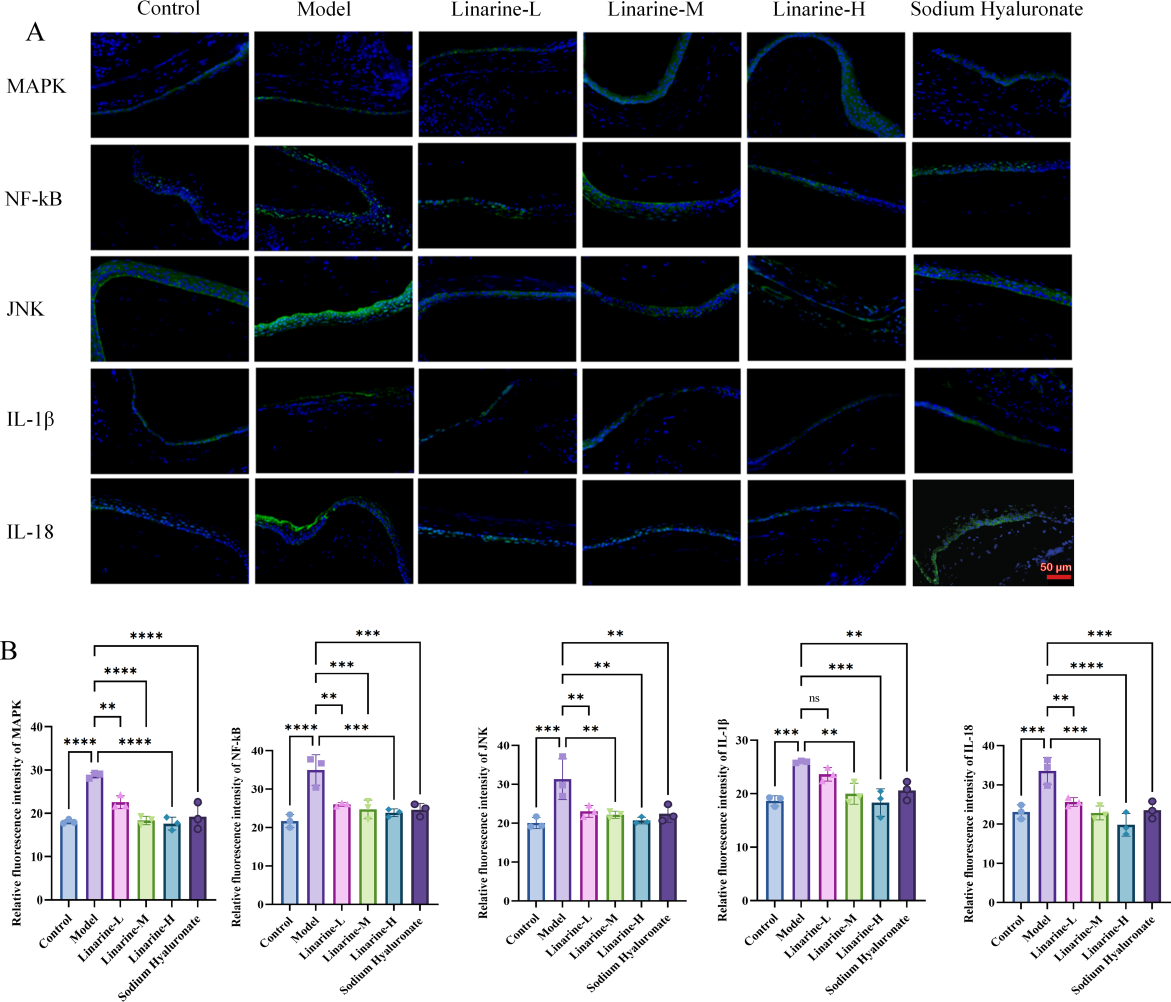
Effect of linarine on the inflammation-related proteins. **(A)** IF of MAPK, NF-kB, JNK, IL-1β, IL-18, and **(B)** Fluorescence quantitative analysis. Values are mean ± SD from 3 samples. ns *P* > 0.05, **P < 0.01,***P < 0.001, ****P < 0.0001 compared with model group.

The modulatory effects of linarine on inflammatory factors were investigated using a mouse model. Compared with the control group, the expression of MAPK, NF-kB, JNK, IL-1β, and IL-18 in the cornea was significantly enhanced in the model group (*P* < 0.01). After the treatment, the expression in the linarine-H group decreased (*P* < 0.01) (**Figures 7A, B**). This suggests that linarine reduces the inflammatory response by inhibiting the production of inflammatory factors.

### 3.7 Effect of linarine on liver, heart, spleen, lung and kidney

**Figure 8:**
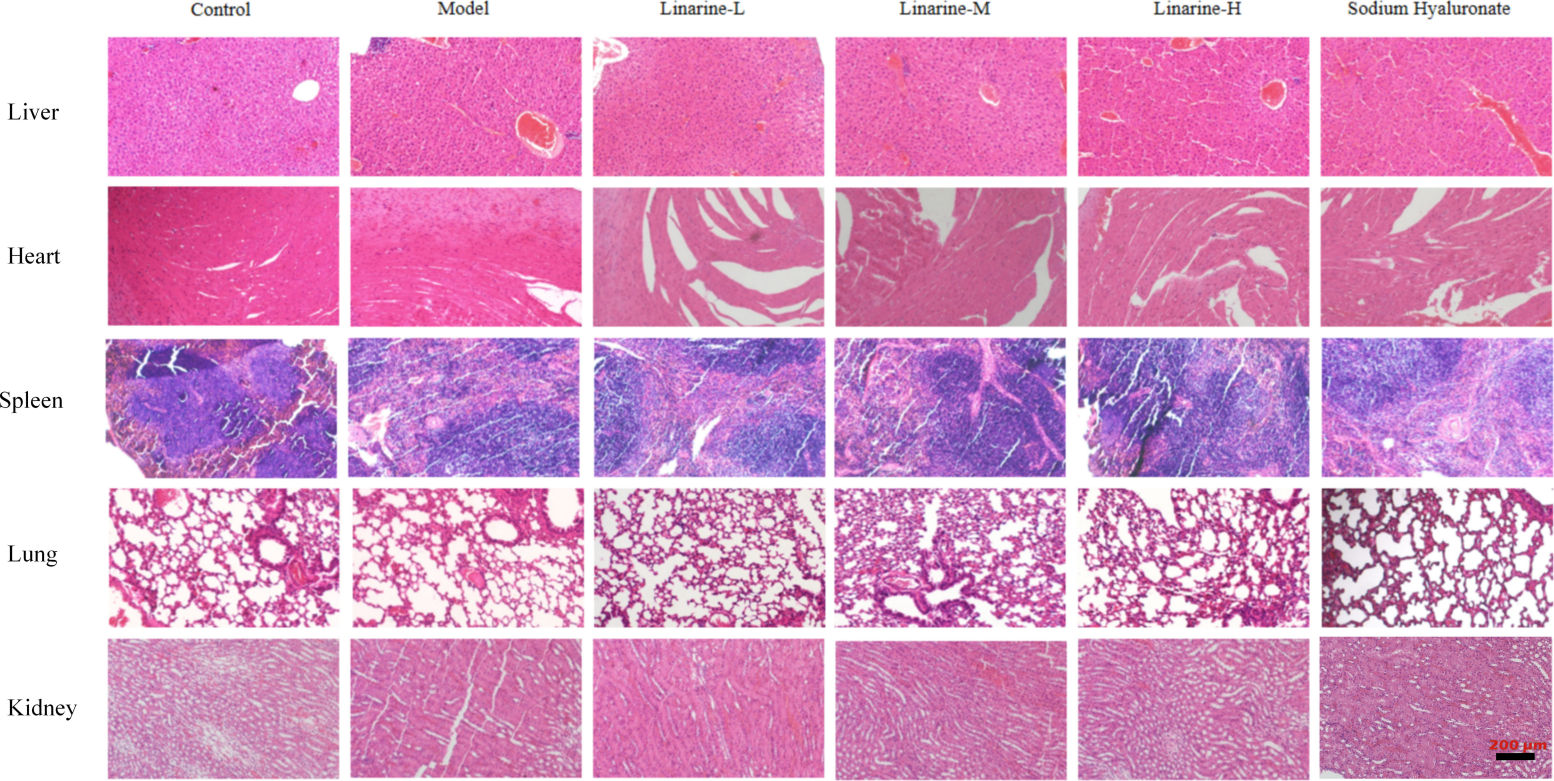
Safety evaluation of linarine *in vivo*. H&E-stained sections of heart, liver, spleen, lung, and kidney tissues from normal control mice and dry eye mice of different treatment groups (n=3).

Determine whether the drug has caused toxicity to the target organ by tissue structural integrity and cellular morphology. (Liver: examine hepatocytes for swelling, necrosis, steatosis, and inflammatory cell infiltration. Observe the structural integrity of the hepatic lobules for hepatic tubular dilatation or fibrosis. Heart: observe cardiac muscle fibers for changes such as necrosis, inflammation and interstitial edema. Assess structural integrity and morphological changes in cardiomyocytes. Spleen: Assess for normal splenic microsomes and lymphocytic infiltration or necrosis of the splenic parenchyma. Examine the structure of the white and red medullas and their alterations. Lungs: observe the alveoli for changes such as edema, inflammation and interalveolar fibrosis. Assess the airways for inflammation or injury. Kidneys: examine the renal units for tubular injury, interstitial inflammation, and tubular epithelial cell necrosis. Observe for dilated renal tubular lumen and filling of renal tubular lumen with abnormal material). HE-stained sections of the liver, it was observed that linarine did not cause significant hepatocyte damage or inflammatory response, and hepatocyte structure and function appeared normal. Cardiac tissues showed that linarine did not cause obvious structural abnormalities or apoptosis in cardiomyocytes, and cardiac function remained good. Splenic sections showed no significant structural changes in splenic vesicles or abnormal cellular responses caused by linarine. Sections of lung tissue showed that linarine did not cause thickening of alveolar walls, inflammatory cell infiltration or other significant pathological changes. Kidney sections showed that linarine did not cause structural changes in the glomeruli or tubules, and renal function was maintained (**Figure 8**). The effects of linarine on these organs showed safety, with no significant tissue damage or functional abnormalities observed.

## 4 Discussion

In clinical practice, dry eye is closely related to several factors. Reduced tear production directly contributes to ocular dryness and discomfort, while a shortened tear film break-up time (TBUT) indicates tear film instability, further exacerbating dry eye symptoms. The presence and extent of corneal damage can reflect the severity of dry eye, while elevated inflammatory factors such as IL-1β and TNF-α indicate increased ocular inflammation. Dry eye is usually accompanied by an inflammatory response in ocular tissues. Purinergic receptors are a class of proteins that recognize and respond to purine-like compounds and have important roles in immune responses and inflammatory processes (11). It was reported that purinergic receptors may be involved in regulating the release of inflammatory mediators and the activation of inflammatory cells, thereby influencing the development and progression of dry eye (6).

Purinergic receptors, consists of the P1 receptor and P2X receptors and P2Y receptors. P1 receptor can be divided into four subtypes of adenosine A1, A2A, A2B, and A3, while P2X receptors include subtypes of P2X1-7, and eight subtypes of P2Y receptors: P2Y1, P2Y2, P2Y4, P2Y6, and P2Y11-14, of which the A1, A2A, A3, P2X4, P2X7, P2Y1, P2Y2 and P2Y4 receptors play important regulatory roles in the inflammatory response in dry eye (6). The A2A receptor in the P1 receptor mediates the inflammatory response in dry eye mainly by regulating the activation of the MAPK/NF-kB pathway. This kind of receptor subtype presenting in most cells is a key kinase in most signaling pathways, and is mainly involved in the inflammatory response (12). As one kind of subtype of P2 receptors, the P2X4 receptor promotes receptor-associated activation of proinflammatory cytokines and inflammatory vesicles, which mainly activates NLRP3 inflammasome, the secretion of IL-1β and promotes conjunctival inflammation through the caspase-1 pathway (13). The P2X7 receptor promotes the activation of inflammasomal vesicles and the release of proinflammatory cytokines IL-1β and IL-18. P2X7 receptor can promote the activation of inflammatory vesicles and the release of pro-inflammatory cytokines IL-1β and IL-18 (14, 15). P2X7 receptor also decreased the intracellular K+,while increase in cytoplasmic Ca2+ ions, which can activate caspase-1, leading to the rapid activation and release of pro-inflammatory cytokine IL-1β (16–18). Nitric oxide synthase, cyclooxygenase-2, TNF-α, phospholipase D, phospholipase A2, NF-kB, and MAPK (17, 18), which ultimately leads to dry eye.

Commonly used anti-inflammatory drugs for dry eyes include corticosteroids, tetracycline derivatives, non-steroidal anti-inflammatory drugs (NSAIDs), and autologous serum (19). However, long-term use of corticosteroids is prone to side effects such as elevated intraocular pressure and corneal infections. Even though tetracycline derivatives have fewer side effects compared to corticosteroids, patients need to be closely monitored during their use. However, NSAIDs also have some side effects, including gastrointestinal discomfort and allergic reactions. Linarine is a natural flavonoid compound mainly found in plants such as wild chrysanthemum and mimosine, which has antibacterial, anti-inflammatory, antioxidant and other pharmacological effects. Moreover, it has no obvious toxic side effects, basically have no effect on the kidney and liver, and the therapeutic effect is precise, safe and controllable, which has high practical application value (9).

In this study, we found that linarine significantly inhibited the inflammatory response of the ocular surface in an animal model of dry eye by *in vitro* and *in vivo* experiments. Corneal epithelial cells, as an important component of the ocular surface, are often affected by injury and metabolic disorders in dry eye. Linarine may promote the repair and regeneration of corneal epithelial cells through its anti-inflammatory and cytoprotective effects. This was partially verified in cellular experiments, where linarine reduced the expression of TNF-α and IL-1β in a corneal epithelial cell inflammation model. We performed further molecular mechanism studies by animal experiments. Linarine increased body weight and lowered body temperature in dry eye model mice, significantly prolonged tear film rupture time, promoted tear secretion, repaired corneal damage, and reduced the expression levels of inflammatory factors MAPK, NF-kB, JNK, IL-1β, and IL-18, thereby reducing the severity of inflammatory response. Linarine reduces the symptoms of dry eye by modulating inflammatory pathways through multiple mechanisms. Specifically, it reduces the inflammatory response by inhibiting the activation of MAPK (including JNK and p38 MAPK, etc.). The MAPK pathway plays an important role in the inflammatory process, with JNK being associated with cellular stress and apoptosis, and p38 MAPK being closely associated with stress response and inflammation. By inhibiting the phosphorylation of these MAPKs, linarine reduces the levels of pro-inflammatory cytokines such as IL-1β and IL-18, which play a central role in inflammation. On the other hand, linarine also inhibits the activation of the NF-κB signaling pathway and reduces the expression of pro-inflammatory factors, a key transcription factor that regulates the expression of a variety of inflammation-related genes. By integrally regulating these signaling pathways, linarine effectively reduces the inflammatory response, thereby improving the symptoms of dry eye, such as dryness, irritation and redness of the eyes.

The role of purinergic receptors in inflammatory responses is widely recognized, our experimental results also show that linarine modulates the expression and activity of purinergic receptors. Linarine regulates A2A receptor activity, which normally inhibits pro-inflammatory responses and reduces cytokine production. By affecting the A2A receptor, linarine enhances anti-inflammatory effects and reduces tissue damage in diseases such as dry eye. Linarine interacts with the A3 receptor to regulate its activity. A3 receptor activation is protective against inflammation. Linarine may enhance this protective effect, reducing inflammation and promoting tissue repair. Linarine’s action on P2X4 receptors may help modulate the damage perception pathway, thereby reducing pain and decreasing inflammatory symptoms associated with dry eye. Linarine may inhibit P2X7 receptor activity, thereby reducing the release of these inflammatory mediators and reducing inflammation. By modulating P2Y1, Linarine may help reduce inflammation and improve ocular surface health. Linarine reduces inflammatory responses on the ocular surface by modulating these receptors, thereby reducing dryness and discomfort. Reduces pain and improves overall comfort. Reduces damage to the ocular surface and promotes healing. In the linarine-treated group, there were significant changes in the expression levels of A2A, A3, P2X4, P2X7, and P2Y1 receptors, suggesting that linarine can reduce the release of inflammatory factors by inhibiting the activity of purinergic receptors, thereby improving the inflammatory state of the ocular surface. Taken together, we hypothesize that linarine play an anti-inflammatory role by inhibiting purinergic receptor activity, thereby reducing corneal epithelial damage and improving dry eye symptoms.

Although this study demonstrates the potential effects of linarine on inhibiting dry eye inflammation and purinergic receptors, its application still faces some potential limitations and challenges. First, as a natural compound, the pharmacological properties and safety of linarine have been preliminarily validated in several disease models, but its metabolism, pharmacokinetics, and safety for long-term use in humans still need to be further evaluated. In particular, more clinical trials are needed to confirm the efficacy of its long-term use in the long-term treatment of patients with dry eye.

In addition, although this study focused on the modulatory effects of linarine on purinergic receptors, the roles, and interrelationships of different types of receptors in the purinergic receptor family need to be investigated in detail. In particular, the specific regulation of receptor subtypes and the expression differences in different cell types remain to be further explored.

## Data Availability

The original contributions presented in the study are included in the article/supplementary material. Further inquiries can be directed to the corresponding authors.

## References

[1] Wirta D. Update on dry eye disease. Jama-Journal Am Med Assoc. (2022) 327:2355–6. doi: 10.1001/jama.2022.6369 35727283

[2] Safir M Twig G Mimouni M. Dry eye disease management. Bmj-British Med J. (2024) 384:e077344. doi: 10.1136/bmj-2023-077344 38527751

[3] Rhee MK Mah FS. Inflammation in dry eye disease. Ophthalmology. (2017) 124:S14–9. doi: 10.1016/j.ophtha.2017.08.029 29055357

[4] Wei Y Asbell PA. sPLA-IIa participates in ocular surface inflammation in humans with dry eye disease. Exp Eye Res. (2020) 201:108209. doi: 10.1016/j.exer.2020.108209 33011237

[5] Zheng QH. Radioligands targeting purinergic P2X7 receptor. Bioorganic Medicinal Chem Lett. (2020) 30:127169. doi: 10.1016/j.bmcl.2020.127169 32273217

[6] Wang JN Fan H Song JT. Targeting purinergic receptors to attenuate inflammation of dry eye. Purinergic Signalling. (2023) 19:199–206. doi: 10.1007/s11302-022-09851-9 35218451 PMC9984584

[7] Ling JW Chan BP Tsang M S-M Gao X Leung PC Lam C W-K. Current advances in mechanisms and treatment of dry eye disease: toward anti-inflammatory and immunomodulatory therapy and traditional chinese medicine. Front Med. (2022) 8. doi: 10.3389/fmed.2021.815075 PMC8801439 35111787

[8] Zhang L Sun L Gao S Yang W Zhuang Y Xu M. Buddleoside inhibits progression of liver cancer by regulating NFκB signaling pathway. Lett Drug Design Discov. (2024) 21:166–73. doi: 10.2174/1570180820666230308115303

[9] Cao JF Wu C Ye W Yang Z Chang Z. Buddleoside inhibits TLR4-related pathway in a mouse model of acute liver failure, promotes autophagy, and inhibits inflammation. Trop J Pharm Res. (2022) 21:515–20. doi: 10.4314/tjpr.v21i3.9

[10] Liu P Jiang PF Yu Y Tan K Qin G Liu T. Modified Danzhi Xiaoyao Powder (MDXP) improves the corneal damage in dry eye disease (DED) mice through phagocytosis. J Ethnopharmacology. (2024) 321:117544. doi: 10.1016/j.jep.2023.117544 38070838

[11] Eltzschig HK Sitkovsky MV Robson SC. Purinergic signaling during inflammation REPLY. New Engl J Med. (2013) 368:1260–0. doi: 10.1056/nejmc1300259 23534573

[12] Wen XM Jiao LD Tan H. MAPK/ERK pathway as a central regulator in vertebrate organ regeneration. Int J Mol Sci. (2022) 23:1464. doi: 10.3390/ijms23031464 35163418 PMC8835994

[13] Sosne G Rimmer D Kleinman HK Ousler G. Thymosin beta 4: A potential novel therapy for neurotrophic keratopathy, dry eye, and ocular surface diseases. Thymosins. (2016) 102:277–306. doi: 10.1016/bs.vh.2016.04.012 27450739

[14] Puro DG. Impact of P2X7 purinoceptors on goblet cell function: implications for dry eye. Int J Mol Sci. (2021) 22:6935. doi: 10.3390/ijms22136935 34203249 PMC8267735

[15] Puro DG. How goblet cells respond to dry eye: adaptive and pathological roles of voltage-gated calcium channels and P2X purinoceptors . Am J Physiology-Cell Physiol. (2020) 318:C1305–15. doi: 10.1152/ajpcell.00086.2020 PMC7311746 32348177

[16] Guzman-Aranguez A de Lara MJP Pintor J. Hyperosmotic stress induces ATP release and changes in P2X7 receptor levels in human corneal and conjunctival epithelial cells. Purinergic Signalling. (2017) 13:249–58. doi: 10.1007/s11302-017-9556-5 PMC5432484 28176024

[17] Li LL Jasmer KJ Camden JM Woods LT Martin AL Yang Y. Early dry eye disease onset in a NOD.H-2h4 mouse model of sjogren's syndrome. Invest Ophthalmol Visual Sci. (2022) 63:18. doi: 10.1167/iovs.63.6.18 PMC9233292 35727180

[18] Dartt DA. P2X7 receptors in the lacrimal gland: Role in health or disease? Invest Ophthalmol Visual Sci. (2019) 60:4205.31618424

[19] Nagai N Otake H. Novel drug delivery systems for the management of dry eye. Advanced Drug Delivery Rev. (2022) 191:11458. doi: 10.1016/j.addr.2022.114582 36283491

